# Assessing factors influencing adolescents’ dietary behaviours in urban Ethiopia using participatory photography

**DOI:** 10.1017/S1368980020002487

**Published:** 2021-08

**Authors:** Ursula Trübswasser, Kaleab Baye, Michelle Holdsworth, Megan Loeffen, Edith JM Feskens, Elise F Talsma

**Affiliations:** 1 Division of Human Nutrition and Health, Wageningen University, Wageningen, The Netherlands; 2 Center for Food Science and Nutrition, Addis Ababa University, Addis Ababa, Ethiopia; 3 Food and Nutrition in the Global South Research Unit (NUTRIPASS), IRD – Institut de Recherche pour le Développement, Montpellier, France

**Keywords:** Adolescents, Dietary behaviour, Food environment, Photovoice, Africa

## Abstract

**Objective:**

To assess factors influencing dietary behaviours of adolescents in Addis Ababa, Ethiopia.

**Design:**

Using the qualitative participatory method Photovoice, participants received training on the basics of Photovoice and took photographs related to (un)healthy eating in their environment. Transcripts of individual interviews, focus group discussions and photographs were coded for thematic analysis.

**Setting:**

One private and one public school located in the same, central neighbourhood in Addis Ababa, Ethiopia to explore how school populations of different socio-economic status experience the same neighbourhood environment.

**Participants:**

Twenty-six adolescents aged 14–19 years old, of which there were seventeen girls and nine boys.

**Results:**

Findings from the current study indicate that food safety concerns appear to be the major influencing factors for adolescents’ dietary choices. Unhealthy and unsafe foods appear to be widely available and/or affordable in adolescents’ neighbourhoods and almost half of the photographs taken by adolescents depicted poor hygiene conditions related to food vendors. Participants considered foods available in their environments as generally unsafe, calling for more packaged food.

**Conclusions:**

Concerns for food safety, hygiene and affordability are the dominating factors for adolescents’ food choices. These concerns, together with limited nutrition knowledge and preference for packaged foods, could make cheap, ultra-processed packaged foods more desirable.

Globally, more children and adolescents are moderately or severely underweight than obese, but obesity is expected to overtake underweight rates by 2022^(1)^. Rates of overweight and obesity in 12–15 year olds in low- and middle-income countries (LMIC) have been reported at 21·4 %^(2)^.

Most research in recent years in LMIC has targeted maternal and child nutrition, aiming to interrupt the intergenerational cycle of malnutrition^(3)^. More recently, adolescence has been identified not only as a second window of opportunity for catch-up growth^(4)^ but also as a period in which nutritional needs increase and lifelong dietary habits and preferences are formed. These habits can influence adolescents’ nutritional status as well as that of future generations^(5)^.

The link between poor-quality diets low in diversity with malnutrition has been well established^(6)^. However, little research in LMIC has been conducted on the factors influencing dietary choices^(7–9)^. Some of these factors are related to increasing urbanisation and changes in urban food environments, encompassing food availability, physical and economic access; advertising; and food quality and safety^(10)^. Increased availability of energy-dense, nutrient-poor, ultra-processed foods is potentially detrimental to adolescent diets and nutritional status, as research from LMIC has shown^(8,11)^.

Ethiopia has a rapidly growing urban population^(12)^ that is increasingly spending more on animal products, oils and fats, as well as fruit and vegetables, while prices for sugar and oil have been decreasing^(13)^. However, overweight currently affects <5 % of adolescents and is mostly a concern for adult women in urban populations of higher wealth quintiles in Ethiopia^(14)^. As research from other LMIC has shown, these trends are likely to shift to other age and socio-economic groups^(11)^.

For adolescents in particular, unhealthy school food environments, as well as parents and peers, can influence their food choices^(15,16)^. Assessing adolescents’ views of their food environment could provide a better understanding of potential factors influencing their food choices^(17)^. This could further contribute to knowledge related to factors of the personal food environment, such as desirability, affordability and accessibility, which remain understudied^(10)^. In addition, the use of qualitative research in LMIC has been identified as a gap in adolescent research^(15,18)^. Photovoice is a participatory action research methodology that has been used previously in food environment studies with adolescents in resource-poor settings of high-income countries or in LMIC^(17,19–21)^. This Photovoice study will therefore fill a significant gap in understanding the factors influencing dietary behaviours of adolescents in the urban LMIC setting of Addis Ababa, Ethiopia.

## Methods

### Participants and setting

The current study was conducted in two schools in Addis Ababa, Ethiopia. Schools are a convenient setting to access adolescents in urban settings for studies that require participants to be gathered on multiple occasions. One private and one government school (GS) were selected based on recommendations from the Bureau of Education of the City of Addis Ababa, based on the commitment of the school principals and proximity to each other. The schools are 1·29 km apart and share a similar neighbourhood in the busy, central part of town, called Sidist Kilo. The selection of school types was used as a proxy for socio-economic status^(22)^. Physical proximity to each other was important to explore how school populations of different socio-economic status experience the same neighbourhood environment.

Based on sample sizes of previous Photovoice studies^(23)^, fifteen adolescents were recruited for each school using purposive sampling with the assistance of one teacher in each school. To be eligible, participants had to be between 14 and 19 years old, own a smartphone and be committed to participate in one meeting each week for the 4 weeks of the study. The age group of 14–19 years was selected as the study methodology required a high level of participation, communication and conceptualisation skills, which older adolescents are more likely to provide^(24)^. In this age group, school attendance in Addis Ababa is higher than 60 %^(25)^. Since the rules of the private school (PS) prohibited students from carrying mobile phones on the school premises, digital cameras (Sony DSCW800) were given to the PS students for the period of the study.

### Design and data collection

The current study used the Photovoice method to assess adolescents’ perceptions of factors influencing their dietary behaviours, following the three-staged approach of *selecting* (choosing the photographs that best reflect the topic); *contextualising* (explaining the context of the photographs) and *codifying* (identifying the issues, themes and theories emerging from the photographs)^(26)^.

Data collection in the GS was conducted during October to November 2018 and in the PS during May to June 2019. At the beginning of the study, one-on-one interviews were conducted with all participants to collect information on their demographic profile including age, gender and residence. After receiving training on the objectives of the study, basic photography, ethics and safety, participants were asked to take photographs during a 2-week period on the following two topics: (1) ‘Challenges in your environment to eat healthy’ and (2) ‘Opportunities in your environment to eat healthy’. Participants were asked to take a minimum of three photographs per topic but were instructed not to undertake any selection of photographs prior to their interview. The training did not include any information on nutrition or definitions of ‘healthy diet’ to avoid influencing the knowledge and perceptions of participants related to ‘healthy diet’. The four facilitators (three females, one male) had prior experience in qualitative research and/or in working with adolescents and were fluent in the local language, Amharic. They were trained by two researchers on the study protocol and facilitation techniques to guide participants during the study period. All questionnaires and materials for participants were translated into Amharic and then back translated into English to ensure accuracy.

When participants returned with their photographs, individual photo-elicit interviews were conducted, during which participants provided captions for every photograph and selected a maximum of three photographs per topic, for a total of no more than six photographs. Facilitators gently guided participants in the selection process by reminding them of the topics. Interviews lasted on average 50 min. The interviews followed a set of interview questions, abbreviated as ‘SHOWeD’ in the Photovoice methodology^(26)^, which were adapted with supporting questions, to make them more understandable for adolescents^(24)^: (supporting questions are provided in parentheses) (1) What do you **S**ee here?; (2) What is really **H**appening here? (What might have happened before the photo was taken?; Why did it happen?); (3) How does this relate to **O**ur lives? (Why did you take the photo and what might others see in the photo?; What did you feel about the subject of the photo?); (4) **W**hy does this problem or this strength exist? (5) Why is the situation like this? and (6) What can we **D**o about this?.

After 1 week, the photographs selected during individual interviews were printed and then used in focus group discussions (FGD). For each school, two FGD were conducted with 5–8 participants and two facilitators in each. In the GS, these FGD were separated by gender; however, in the PS, FGD were conducted in one female-only and one mixed gender group, since there were only two male participants in that school. In the FGD, one photograph was selected for each topic, which was then discussed, following the same ‘SHOWeD’ question guide mentioned above. Combining individual interviews with group discussion enhanced data richness by providing additional insights from individuals on some of the issues raised in the FGD. Individual interviews also provided an opportunity for participants to share more personal reflections, which adolescents may be more self-conscious of doing in a group setting.

### Data analysis

Descriptive data from questionnaires were double entered and checked by a second researcher in Windows Excel and analysed with SPSS Statistics V25.0 for Mac. All interviews and discussions were digitally recorded and simultaneously translated and transcribed from Amharic into English. An *a priori* codebook was developed based on existing literature and structured by four influencing spheres (individual, social, physical and macro-level environment) of a socio-ecological framework^(27)^, which has also been adapted to the African context^(28)^.

All participants were partially involved in data analysis as they selected photographs, identified common themes in the photographs and categorised photographs according to themes. After finalisation of the data collection, two researchers independently conducted first cycle open deductive and inductive coding^(27)^ using ATLAS.ti version 8.3.1 for Mac. The researchers then compared findings, merged codes and examined discrepancies in coding to establish a standardised method^(29)^. Thematic areas were identified by collating data relevant to each code, code group and the four influencing spheres^(30)^.

Furthermore, the 130 photographs selected by participants were categorised inductively based on the different environments (school, neighbourhood, home and social) and different types of foods (fruit and vegetables, fried street food, sugar-sweetened beverages and candy, and home-cooked meals) visible in the photographs. The current analysis of photographs further contributed to identifying themes.

## Results

Out of the thirty recruited adolescents, twenty-six completed the study (female = 17; male = 9). The participants completing the study were aged 14–19 years old and fifteen attended the GS and eleven attended the PS (Table 1). Reasons for loss to follow-up were dropping out of the study (*n* 2) or losing the camera (*n* 2). Participants took a total of 722 photographs (mean = 28), of which they selected 130 photographs (mean = 5; Table 2). Most of the selected photographs (*n* 68) were taken on the streets around the school or the neighbourhoods where the participants lived, which was in the same or neighbouring sub-cities of Addis Ababa to the two schools. The foods pictured in these photographs were primarily of fruit and vegetables sold on the street (*n* 35) or foods prepared on the street, such as fried potatoes, samosas or donuts (*n* 22). The home environment was the second most common environment represented in the photographs (*n* 34), which in some photographs (*n* 22) depicted the home setting, including home-cooked food, family members or food preparations in their kitchens.

**Table 1:** Demographic information of participants in the Photovoice study

Characteristics	*n*	%
Participants’ age (years)	26	
Mean	17·0	
sd	1·8	
School		
Private school	11	42
Public school	15	58
Gender		
Male	9	35
Female	17	65
School year		
9	10	39
11	4	15
12	12	46

**Table 2:** Number of photographs taken by participants, by category

Photo category	*n*	%
Total	722	
Mean	27·8	
sd	21·7	
Selected photographs	130	
Mean	5	
sd	1·3	
Types of food visible		
Street foods	22	17
Fruits or vegetables	35	27
Sugar-sweetened beverages or candy	9	7
Home-cooked food	22	17
Type of environment		
Home setting	34	26
School compound, internal	9	7
Street setting	68	52
Social setting	18	14

### Individual level

#### Knowledge

Knowledge of nutrition and food safety appeared to be an important factor at the individual level. Participants knew about the health benefits of fruit and vegetables, the importance of consuming diverse diets and that certain foods, such as meat and sweet foods, should be consumed in moderation.

**Fig. 1:**
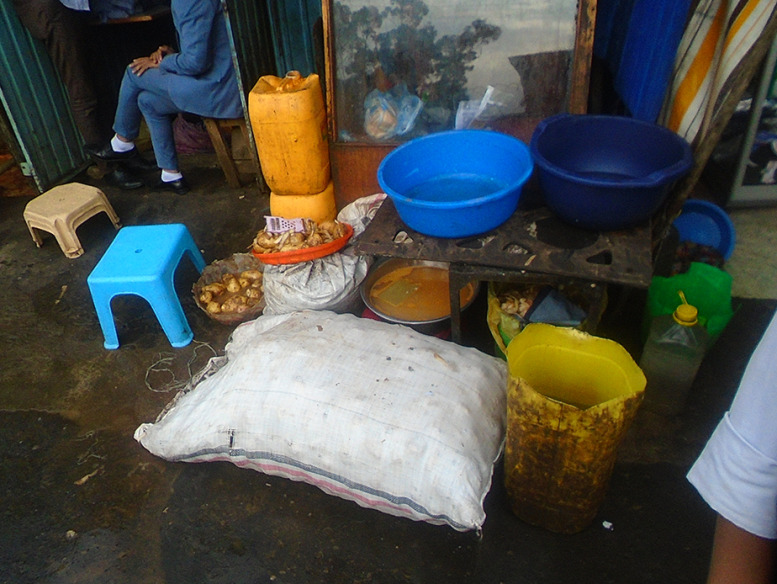
‘Erteb’ (Amharic for potato sandwich) (Girl, 15 years PS)

Participants also had good knowledge related to food safety and hygiene and the resulting effects on health and expressed their concerns about cleanliness. Taking photographs of specific outlets that participants frequented made them further aware of how their food was actually prepared (Fig. 1).

‘…after I do my house chores and my hands get dirty I wash my hands using tap water. Cleanliness is important. Moreover, clean water is important for cooking healthy food.’ (Girl, 16 years, GS)

‘I like potatoes and I would eat it even if you prepare it in any way. And the situation on the picture influences me; it got me thinking is this how our food is prepared?’ (Girl, 15 years PS) (Fig. 1)

The activity where participants assigned photographs to different themes revealed what they perceived as healthy or unhealthy. Photographs assigned to the ‘unhealthy food’ theme included mostly fried foods prepared on the roadside, but also included three photographs of fruit and vegetables sold in carts on the road, which were therefore perceived as unclean. However, fruit and vegetables presented in a clean manner as well as packaged foods sold in shops were associated with ‘healthy foods’.

#### Food preferences

Despite their knowledge, participants liked the taste of fried and sweet foods.

‘I took this picture because most people I know including me consume such sweets and it has become kind of like an addiction.’ (Girl, 17 years, PS)

#### Socio-economic status

Not having the financial means to buy what participants considered healthy food was summarised well by one boy from the GS:

‘It doesn’t matter how knowledgeable we are if that knowledge is not translated into money, then it is useless.’ (Boy, 17 years, GS)

Talking about their own and their families’ financial struggles, participants from the GS expressed how important affordability was in influencing their food choices, and how hard it was for them and their families to make a living.

‘It is the most important factor that influences my food choice, I wish that I could have more money and be able to eat what I want.’ (Girl, 17 years, GS)

Due to these financial limitations, certain foods were considered as outside the participants’ reach, such as meat and fish, while others, such as ‘Shiro’, a legume-based powder that is used for stews or vegetables sold by ‘Gulits’, informal sellers on the side of the road, were considered more available and affordable.

‘Most of us have love for meat and want to eat meat but we can’t afford it.’ (Girl, 18 years, GS)

### Social level

#### Family influence

Families play an important role in adolescents’ food choices, particularly mothers, who do most of the cooking. Mostly in the GS, this influence was perceived as negative, limiting the choices of the adolescents.

‘My mom is the one who cooks everything for us. We have no say in what we eat or want to eat. We have to have what she gives us.’ (FGD, Boys, GS)

Participants from the PS described their mothers’ influence as more positive given their concern for their children’s health.

‘Because mothers usually want the best for their children and want to feed them good food which helps them grow… My mom …doesn’t let me eat raw meat because it would make me sick.’ (Girl, 14 years, PS)

Fathers in the GS were described solely as ‘breadwinners’, while in the PS, fathers appeared to be the ones buying unhealthy foods.

”…my father usually buys me cakes and soft drinks but my mother doesn’t let me have that.” (FGD mixed, PS)

#### Food culture

Participants perceived the Ethiopian tradition of sharing food and eating together as positive and motivating to eat healthy.

‘I grew up eating like this, social events such as this give me the chance to eat healthy.’ (Boy, 18 years, GS)

**Fig. 2:**
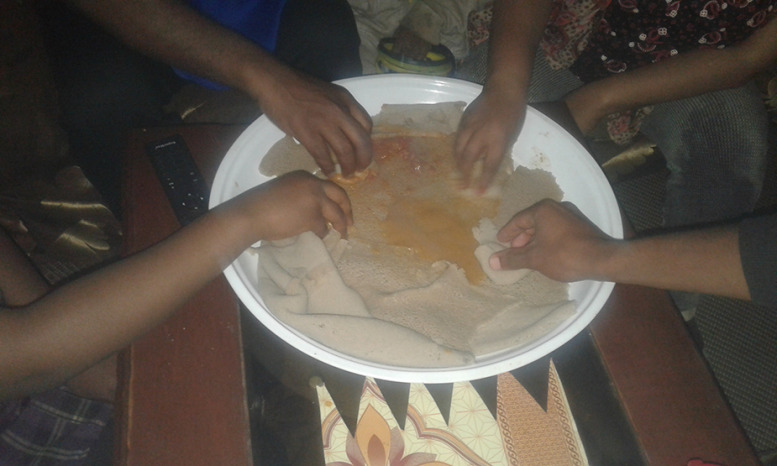
‘Eating together’ (Boy, 18 years, GS)

‘Since I am an Ethiopian I enjoy sharing meals and eating together.’ (Boy, 18 years, GS) – Fig. 2

#### Peer influence

Peer influence was particularly strong in and around the schools, where the adolescents often ate fried street food.

‘My friends like chips and when I’m with them I eat chips too.(…) There is peer pressure around school and peer pressure has the power to change your life and eating at places like this one because friends’ influence might have some impact on my health.’ (Girl, 16 years, PS)

### Physical level

#### Food availability and affordability

All participants were influenced by what is available and affordable in the physical environment around their schools and their homes. The adolescents and their families purchased most of their foods from outlets that were available in their neighbourhoods. There were no supermarkets in the neighbourhoods where they lived, and instead of travelling long distances to supermarkets, the participants and their families bought their food at small kiosks, open markets and informal vendors. Participants, therefore, faced limited options due to lack of affordable outlets forcing them to buy street foods of inadequate quality.

‘Even my friends and I get out of school we sometimes buy and eat this because we don’t have other options.’ (Girl, 15 years, PS)

‘In our neighbourhood this place is our marketplace. This is because we can only afford to buy from here.’ (Boy, 17 years, GS)

#### Hygiene and sanitation

Participants largely categorised outlets into ‘good’ and ‘bad’ based on cleanliness and the way food items were displayed within the shop. When fruit and vegetables were presented in a clean and neat manner, the adolescents were motivated to buy them. Also, if they or their families trusted that the vendor was ‘being careful with the products’, they would prefer doing their food purchases there.

‘Most of us eat fruits and vegetables and the cleanliness of the area and the attractive arrangement is appealing and motivating us to eat healthy food.’ (FGD, girls, GS)

Informal food outlets, such as marketplaces on the sidewalk, were all considered unsafe and unhealthy by the participants. The poor hygiene in these places due to pollution or public urination was a major concern for the participants.

‘“No peeing allowed” so it means people pee over here and the vendors when they come here to set up their items they don’t even clean the area and before they set up people would have peed right there.’ (Boy, 17 years, GS)

When outlets were not clean or the environment around the outlet was dirty, even the sight of such uncleanliness could affect adolescents’ appetites and they reported refraining from buying foods that would actually be beneficial for their health.

‘This is a market place and it is not clean at all. The food sold here might be attractive but because the surroundings are not clean it doesn’t entice you to buy and consume it.’ (Girl, 15 years, PS)

Lack of water in their neighbourhoods as well as their houses could also affect what the adolescents were eating. A participant was concerned about not having water available to wash garlic, which is usually used for stews that are thoroughly cooked.

‘We use garlic in our day-to-day diet. If there’s no water available when we prepare food, we might just peel the garlic and use it without washing it.’ (Boy, 17 years, GS)

#### Packaged food

Given the poor hygiene of the environment, the outlets and the food itself, the participants considered packaged foods as a healthier option. The adolescents also appreciated the information related to ingredients and expiry dates on packaged foods.

**Fig. 3:**
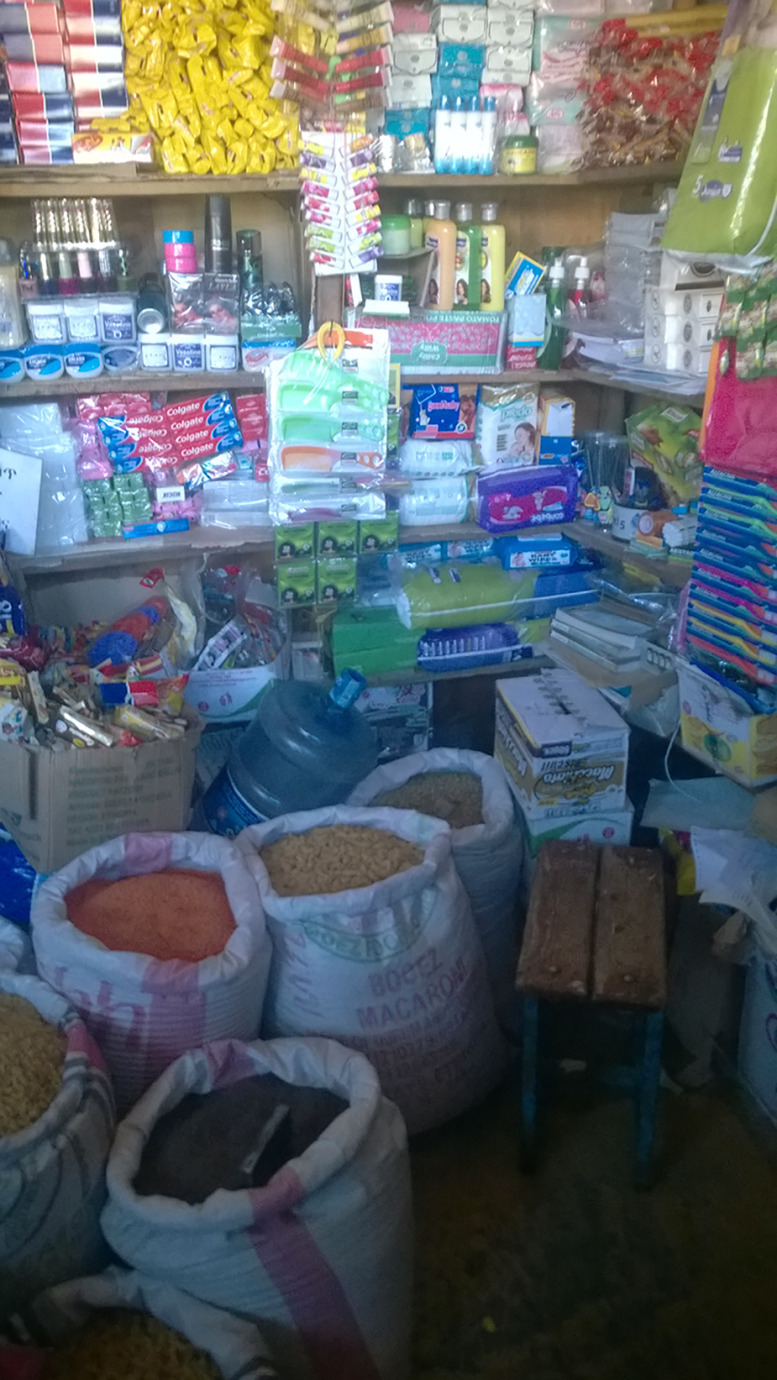
‘The shops supply’ (Boy, 17 years, GS)

‘You can see the packed food here and you can read their contents and understand what you want to eat.’ (Boy 17 years, GS) – Fig. 3

#### Eating out

Eating food that was prepared at home was considered the healthiest, safest and cheapest option for the participants.

‘Home is always better in terms of healthy food instead of eating at restaurants, cafes or from street food. It is well prepared clean and healthy.’ (FGD Boys, GS)

### Macro level

#### Food safety regulations

There were two main areas where participants expressed the need for the government to intervene. The first was related to food safety and poor hygiene of food outlets. The second referred to banning certain food outlets. Since the participants considered the lack of space and financial means of vendors as the main reason for poor hygiene, they believed that the government should enforce food safety guidelines as well as provide appropriate, clean and large spaces for vendors.

‘Hopefully these kinds of places would be improved and blossom into big shops so that they can accommodate more customers and a variety of fresh vegetables.’ (Girl, 14 years, PS)

For some food outlets, particularly the informal vendors selling fruit or vegetables in carts or fried foods outside the school, as well as school canteens, participants called to have them shut down and banned by the government for their lack of cleanliness.

‘I hope to see that street vending and street food become banned in the city.’ (Girl, 16 years, GS)

#### Food prices

The second macro-level issue discussed by participants is related to food prices, which they considered high due to the poor economic situation in the country. Participants also remarked that unhealthy foods like sweets and frying oil were cheap, while foods like meat or fish were rarely affordable.

**Fig. 4:**
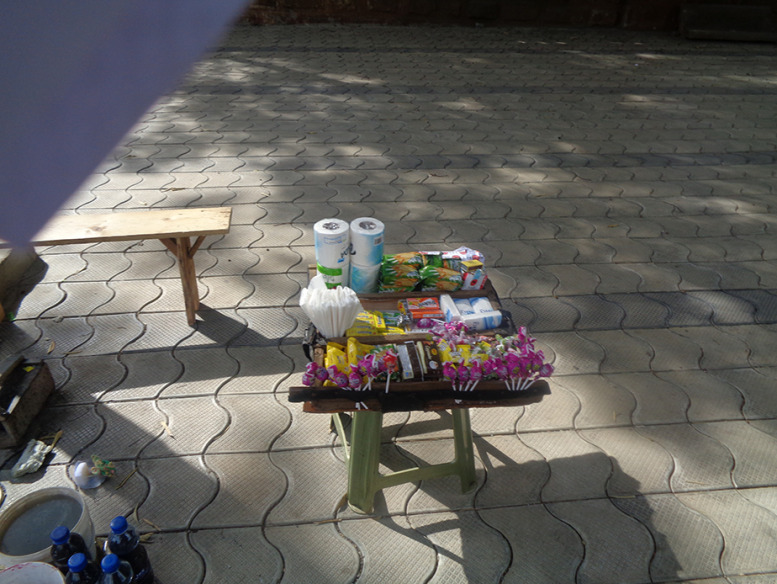
‘Sweet food that we can easily buy’ (Boy, 17 years, GS)

‘Healthy foods should be cheaper and unhealthy foods should become more expensive so people would stop consuming them…. Healthy foods are expensive mostly and we go for cheap stuff sold on the streets. Had both healthy and unhealthy food been the same price we wouldn’t buy cheap food we would have options.’ (Boy, 17 years, GS) – Fig. 4

## Discussion

The current study used participatory photography (Photovoice) to explore the factors influencing dietary behaviours of adolescents in Addis Ababa, Ethiopia. Findings from the photographs, interviews and group discussions undertaken shed light on a number of key factors in adolescents’ environments, particularly in their social and physical environments. While mothers and peers played an important role in adolescents’ food choices, they were mostly influenced by poor hygiene and sanitation of food outlets or their proximal environments. Food prices also appeared to be a key factor for the adolescents attending the GS. However, dietary behaviours of all participants were affected by what is affordable and available in the direct vicinity of their home and school environments.

While participants showed good knowledge related to nutrition, hygiene and food safety, this knowledge did not necessarily translate into their food choices, which could be due to their preference for fried food, possibly related to the taste and appearance of food. Participants further discussed how their choices are limited to what is available and affordable in their neighbourhoods and how they perceived unhealthy foods as cheaper than healthy foods. These observations are supported by previous research from Ethiopia that showed that the price of fruit, vegetables and animal source foods is increasing, while the cost of sugar, oils and fats is decreasing^(13)^.

Since the participants’ knowledge of what is healthy was also skewed towards food safety, their choices were strongly affected by food safety factors in the physical environment. Concerns about the conditions in which foods were prepared or sold and about pollution in general have been reported from other LMIC^(31–33)^. Similarly to the participants in the current study, mothers in a qualitative study in Addis Ababa expressed their fears related to unhygienic street food, while recognising that these foods were also inexpensive^(34)^. Informal sellers and small shops are important in countries like Ethiopia as they often make fruit and vegetables more accessible than supermarkets^(35)^. The participants of the current study suggested banning informal vendors, which could have an impact on the availability of fruit and vegetables^(36,37)^. Unclean food outlets or environments could therefore keep adolescents from eating healthy foods such as fruit and vegetables. Their concerns could push them to consume more packaged food which may include ultra-processed. Lack of water in the households could aggravate this even further due to the fear of not being able to wash fruit and vegetables properly.

Participants in the current study indicated that they had limited decision-making power on the food choices made in their families, particularly by their mother, which is something adolescents also struggle with in other countries^(38)^. However, even when participants were on their way to or from school, where they were free to decide what to eat, they still seemed to choose foods that they themselves consider unhealthy (in nutritional terms). This could be not only due to a preference for fried or snack food^(15)^ but also due to peer pressure^(39)^. While participants perceived the Ethiopian culture of sharing as positive, sharing food with peers could influence them also negatively if group pressure pushes adolescents into consuming unhealthy foods in and around the school^(38,40,41)^.

The application of Photovoice in the current study was useful, not only to identify factors in adolescents’ environments influencing their dietary behaviours, but also because it helped adolescents to record and reflect about their community and to promote critical dialogue about important issues^(42)^. Participants of the current study reported that they appreciated expressing their ‘feelings using photographs rather than talking’, and that the study helped ‘create awareness’ and made them ‘look deeply inside the environment’. Photovoice could be a useful method to give adolescents an opportunity to have their voices heard and to critically look at their environments^(43)^.

While the sample size is appropriate compared with other Photovoice studies^(23)^ and provides important insights into influences of adolescents’ dietary behaviours in LMIC, the findings cannot be generalised to all adolescents in urban Ethiopia. Sampling in the PS was biased towards girls, who were more committed to the study in general, but did not express themselves as much despite the gender-separated FGD. In the GS, the selection criteria of owning a mobile phone could have biased the sample to higher socio-economic groups, even though mobile phone ownership in urban Ethiopia is at 70 %^(14)^.

Application of some SHOWeD questions proved initially to be challenging when photos were taken of issues or food items that did not show an obvious problem. This required the facilitators to follow up with questions to understand the story behind the photograph. Following the first round of individual interviews, the supporting questions were therefore added to the FGD if participants struggled to respond.

The findings of the current study contribute to a better understanding of the factors influencing food choices of adolescents in urban Ethiopia. Adolescents’ knowledge related to nutrition and food safety, their concerns about affordability and hygiene in the physical food environment and food outlets, as well as their families and peers all contribute to their choices. These choices in the long term could also contribute to the disease burden of overweight, obesity and nutrition-related non-communicable diseases in Ethiopia.

Our study identified different ways in which researchers and practitioners could apply the findings in future work. Adolescents in the current study confirmed that they are competent citizens and knowledgeable about their lives, their environment and potential problems in their neighbourhood and are able to identify solutions and should therefore be consulted and involved in future research and programming^(44,45)^. Capitalising on the existing knowledge of adolescents should be key, not only for themselves but also to educate their peers, families or street vendors. Nutrition is currently only integrated into the biology curriculum in Ethiopian schools. Addressing nutrition as part of practical projects across the curriculum, with outreach to parents, vendors and the wider community, would provide an entry point to establish healthy dietary patterns not only for the adolescent but also for other members of the community. Parents should also be closely involved in any nutrition interventions for adolescents, while mothers, as the ones who habitually prepare foods at home, have been targeted traditionally, fathers should also be involved due to their role in food purchasing.

Our findings also identified the need to increase access to healthy, affordable and safe foods in and around schools, which could be achieved through enforcing food safety guidelines for informal sellers^(37)^ and for school canteens^(46)^, taxing unhealthy foods such as sugar-sweetened beverages^(47)^ and/or directly providing or subsidising healthy foods such as fruit and vegetables^(48,49)^. Restricting energy-dense nutrient-poor snack foods from school compounds could help reduce consumption of such foods^(48)^. In addition, the national food-based dietary guidelines that are currently under development could be another opportunity to improve adolescent diets^(50)^. Offering individual pieces of fruit or vegetables around schools has been shown to increase fruit consumption as well as sales of fruit for the vendors^(51)^ and should be considered more widely with free or subsidised schemes within schools.

Future studies should investigate the role that these different interventions could have on the safety, availability and affordability of foods and drinks in urban Ethiopia and how adolescents and their families would respond to them.
